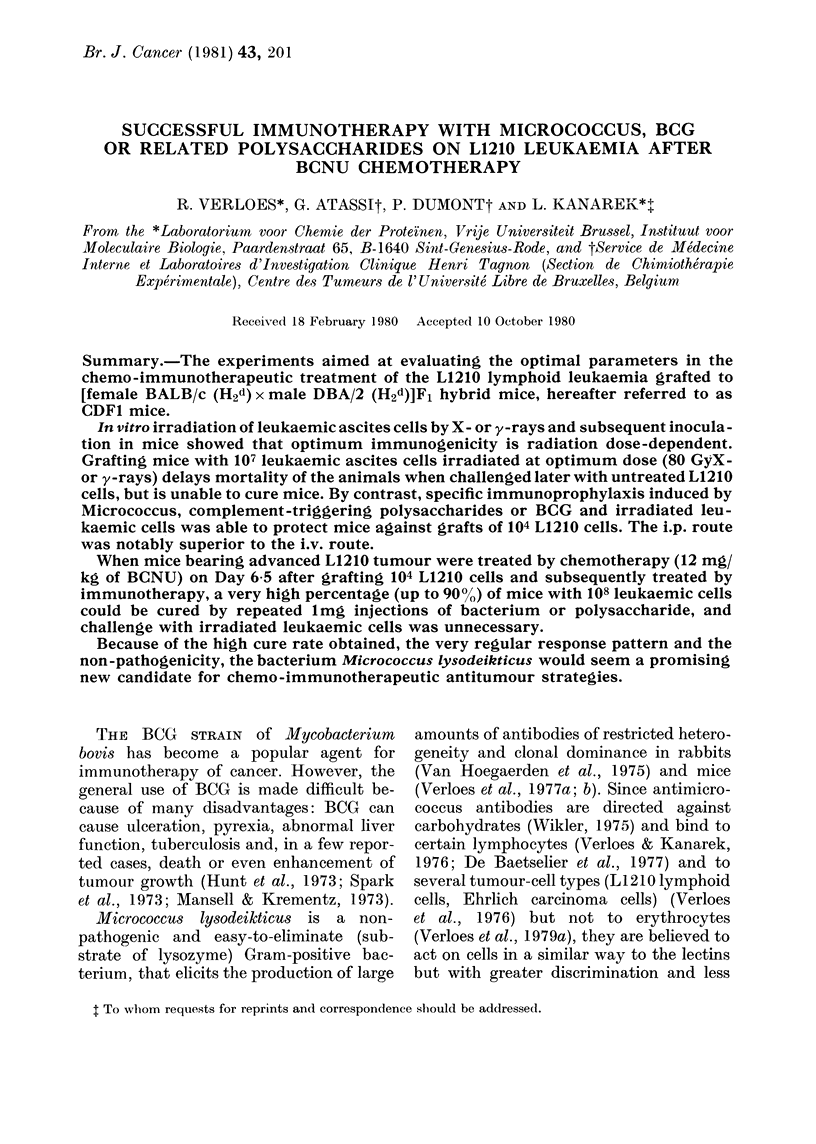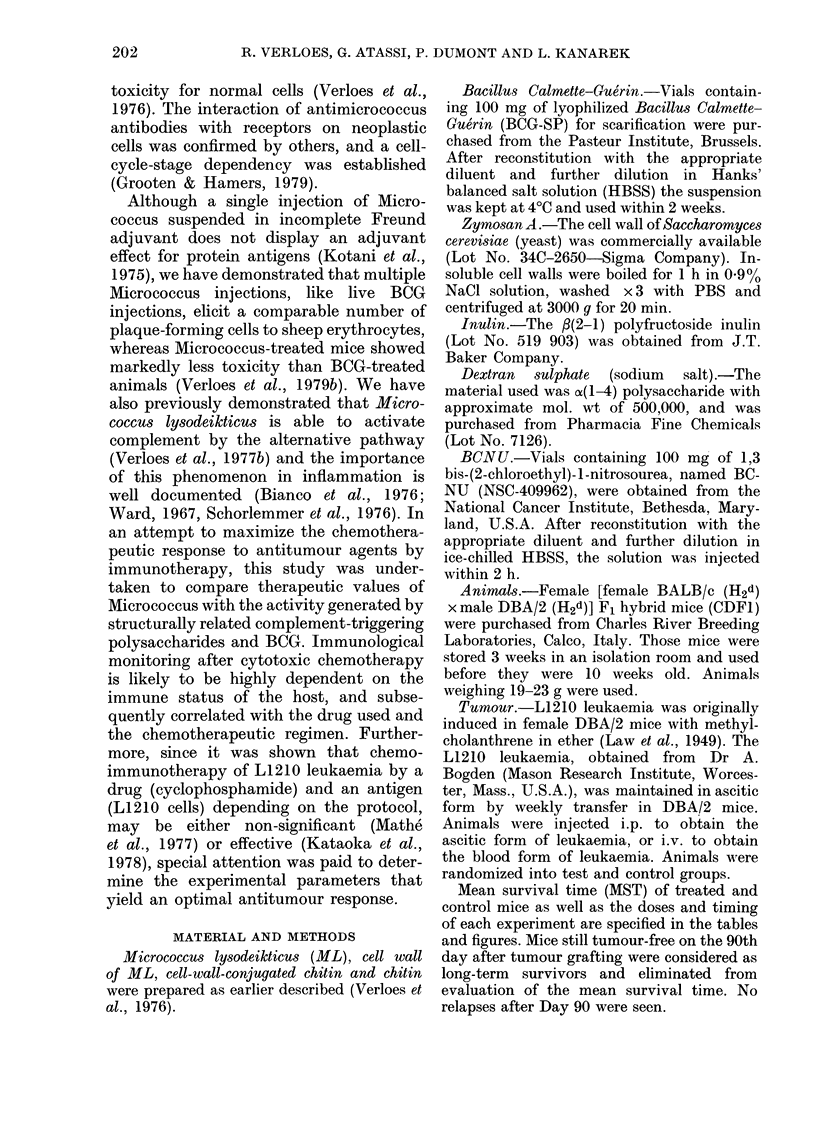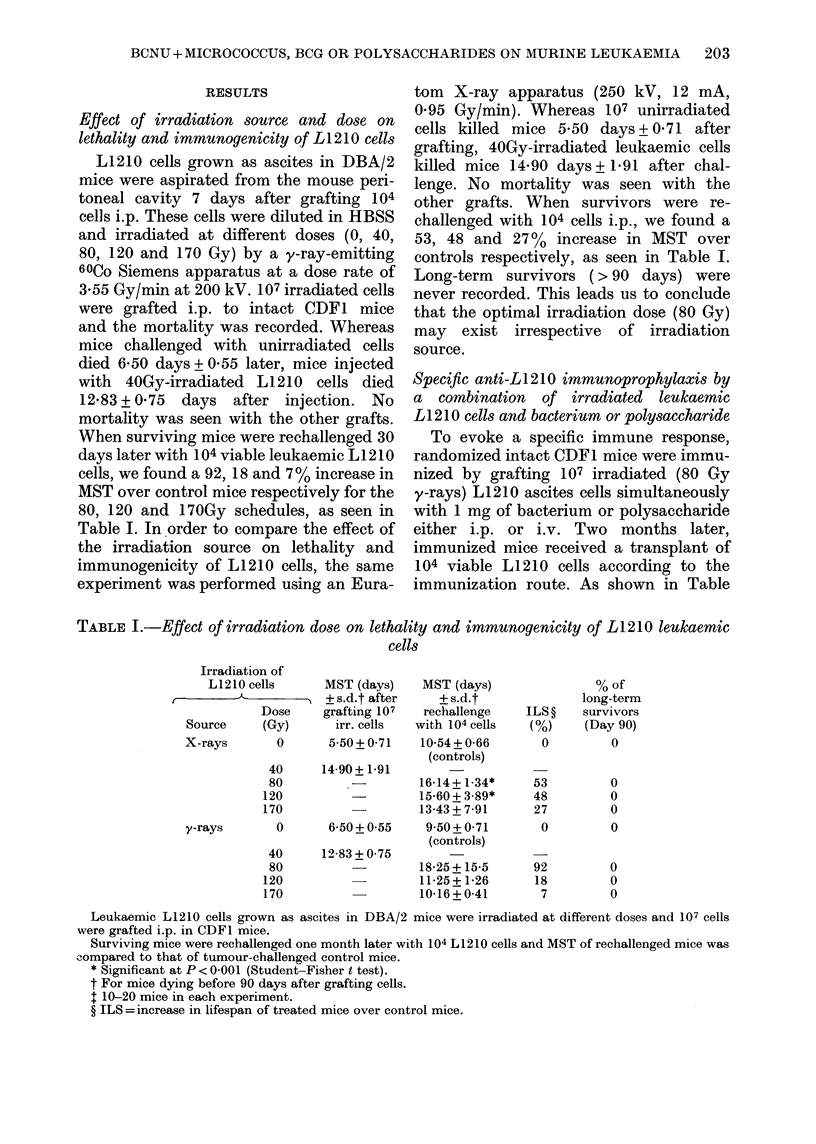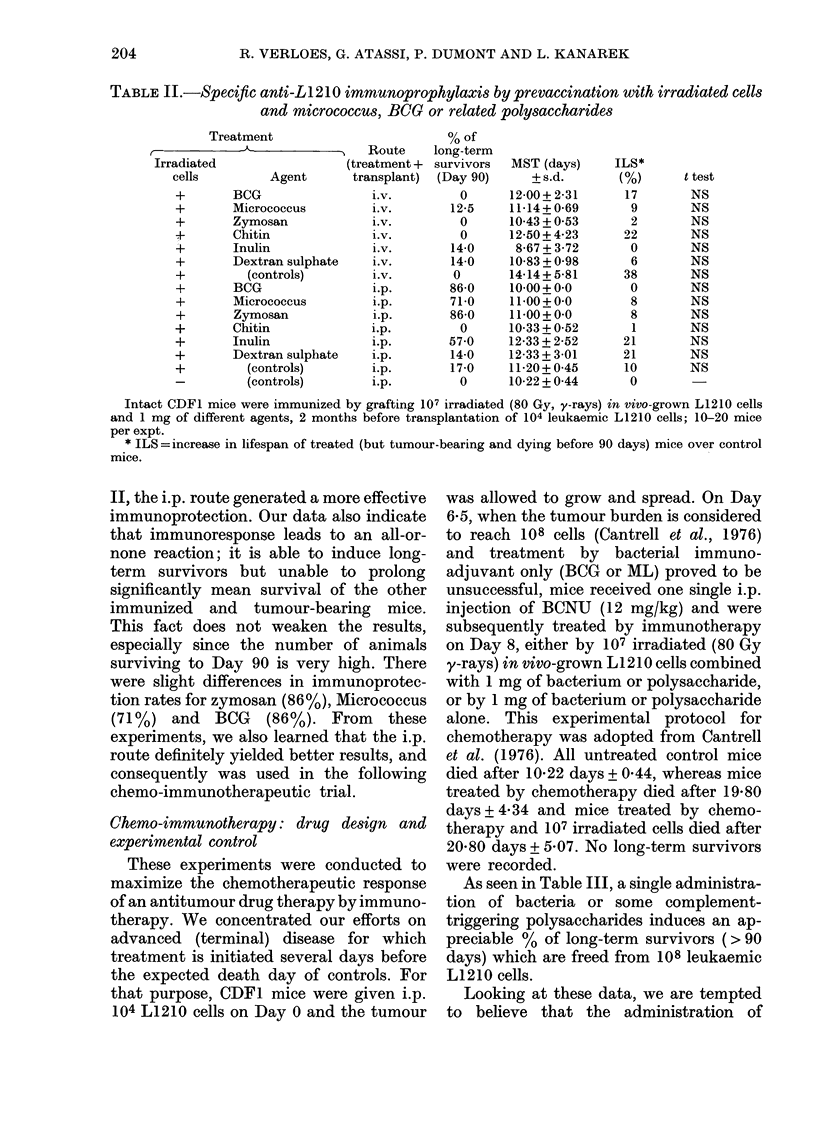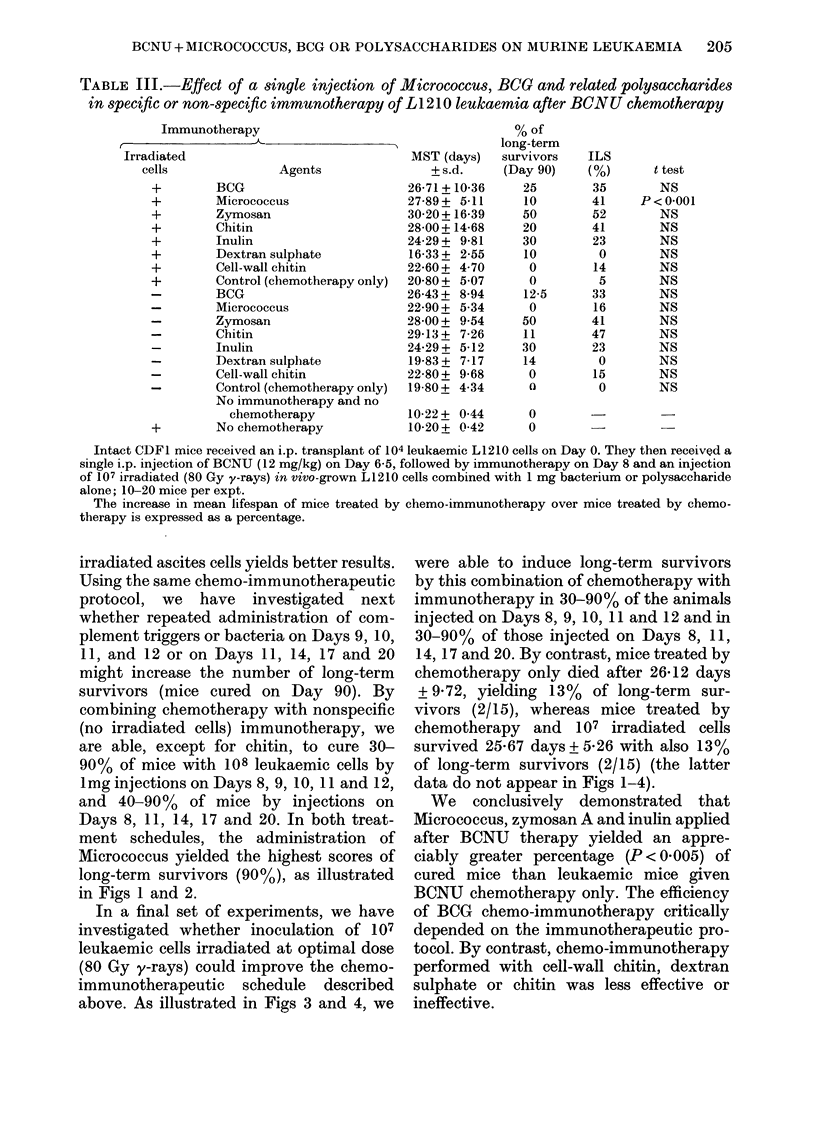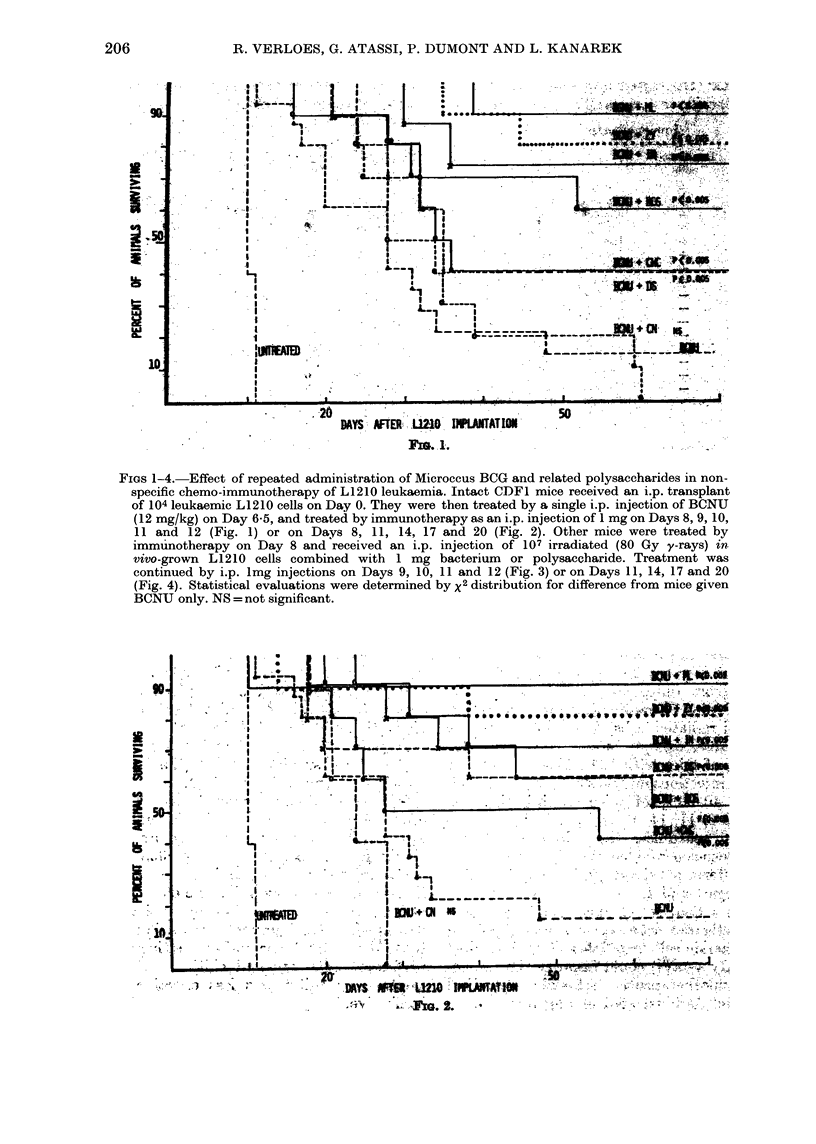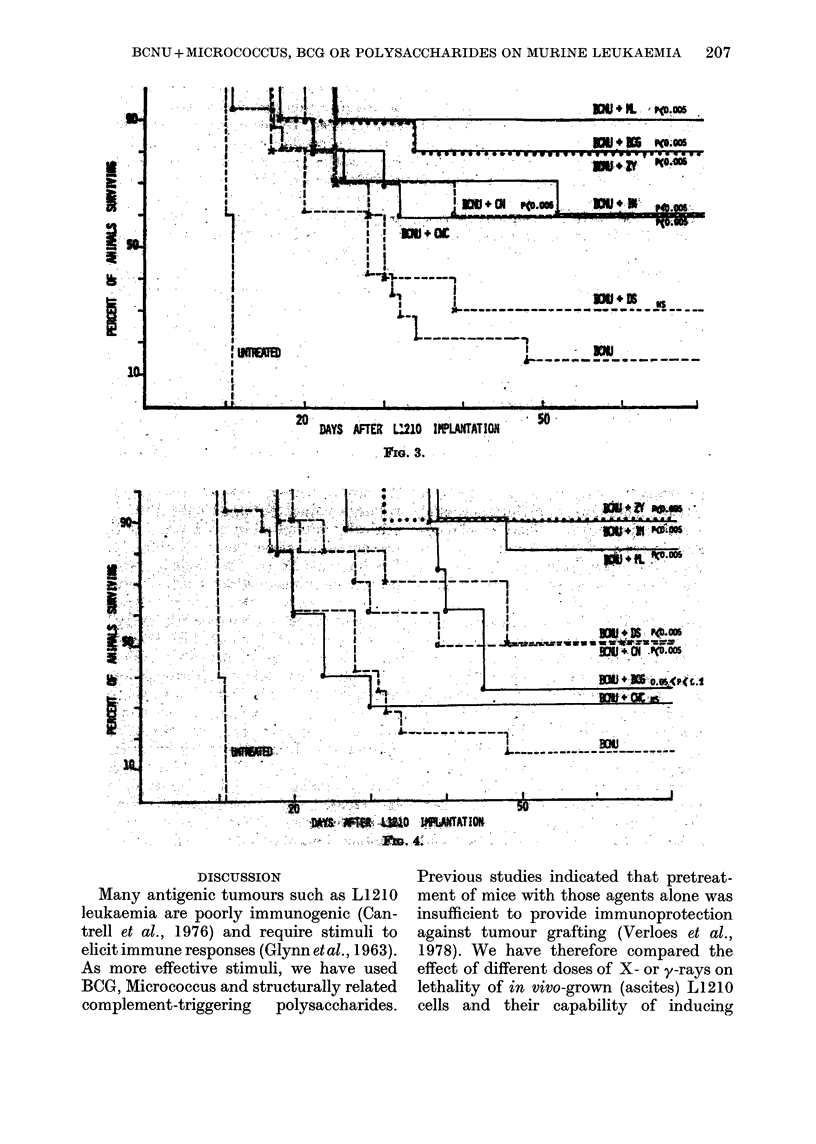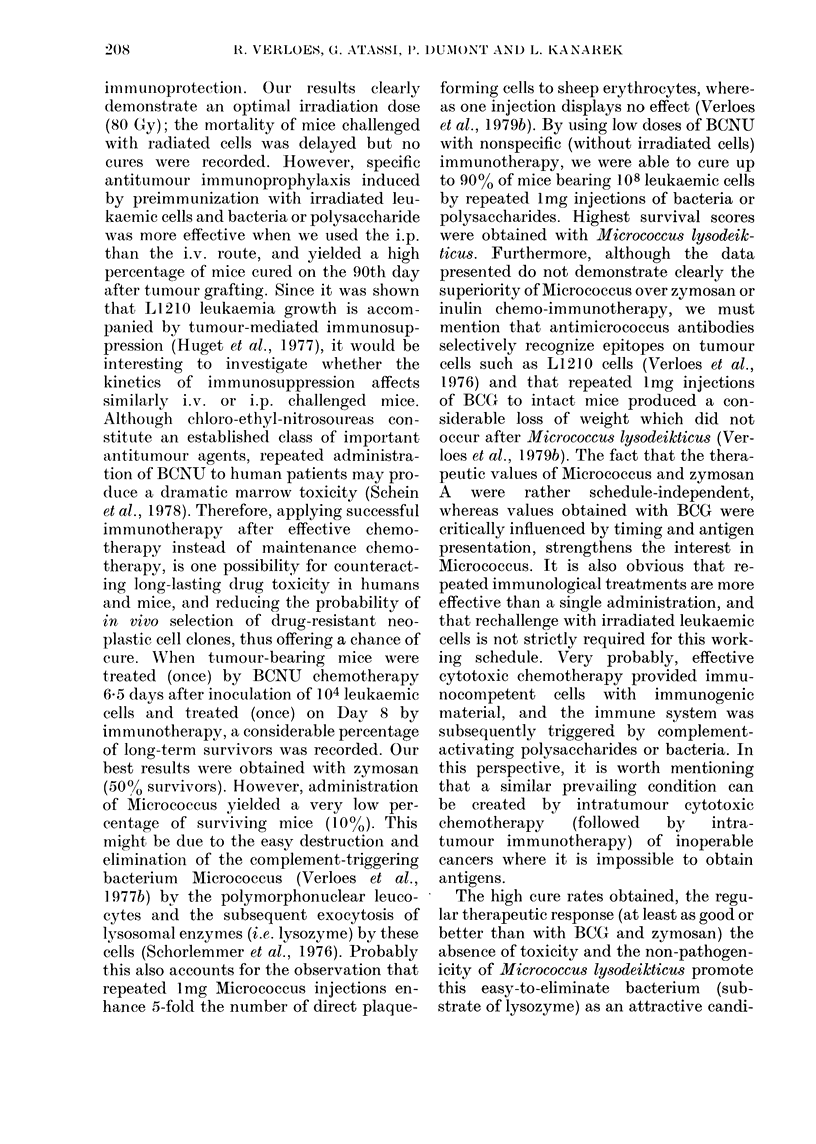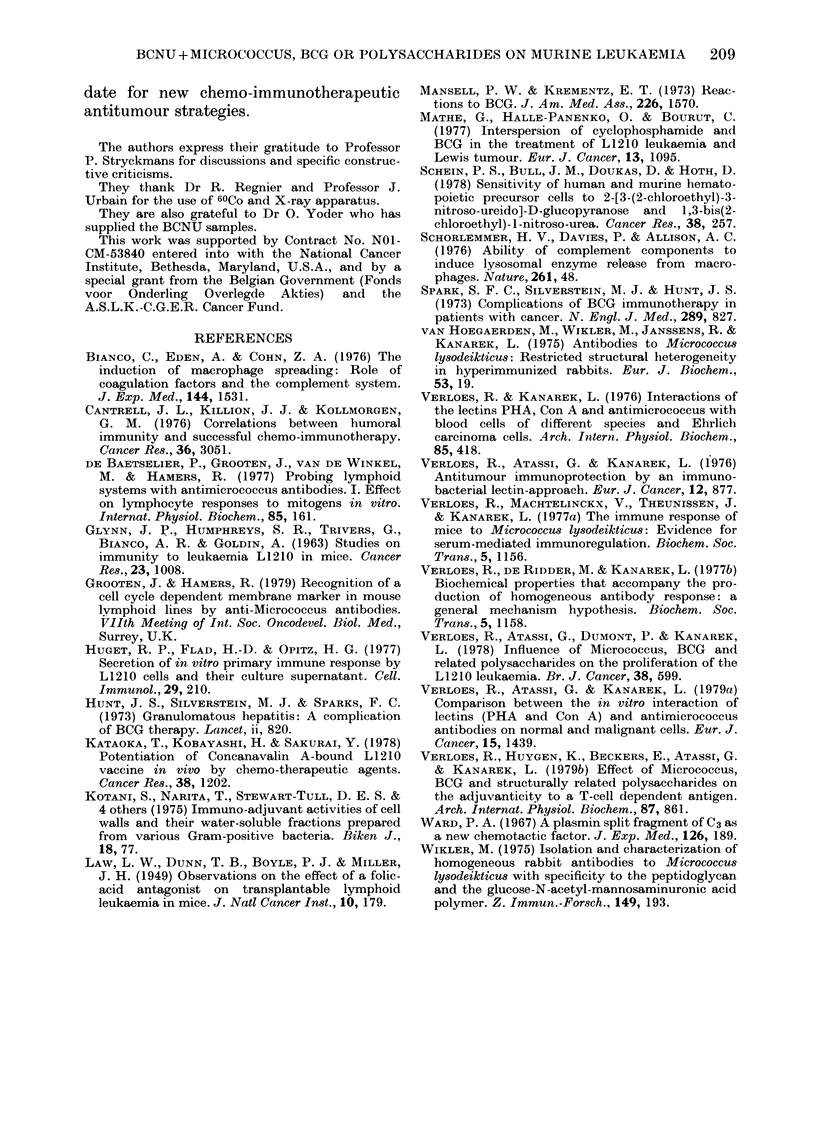# Successful immunotherapy with micrococcus, BCG or related polysaccharides on L1210 leukaemia after BCNU chemotherapy.

**DOI:** 10.1038/bjc.1981.29

**Published:** 1981-02

**Authors:** R. Verloes, G. Atassi, P. Dumont, L. Kanarek

## Abstract

The experiments aimed at evaluating the optimal parameters in the chemo-immunotherapeutic treatment of the L1210 lymphoid leukaemia grafted to [female BALB/c (H2d) X male DBA/2 (H2d)]F1 hybrid mice, hereafter referred to as CDF1 mice. In vitro irradiation of leukaemic ascites cells by X- or gamma-rays and subsequent inoculation in mice showed that optimum immunogenicity is radiation dose-dependent. Grafting mice with 10(7) leukaemic ascites cells irradiated at optimum dose (80 GyX- or gamma-rays) delays mortality of the animals when challenged later with untreated L1210 cells, but is unable to cure mice. By contrast, specific immunoprophylaxis induced by Micrococcus, complement-triggering polysaccharides or BCG and irradiated leukaemic cells was able to protect mice against grafts of 10(4) L1210 cells. The i.p. route was notably superior to the i.v. route. When mice bearing advanced L1210 tumour were treated by chemotherapy (12 mg/kg of BCNU) on Day 6.5 after grafting 10(4) L1210 cells and subsequently treated by immunotherapy, a very high percentage (up to 90%) of mice with 10(8) leukaemic cells could be cured by repeated 1mg injections of bacterium or polysaccharide, and challenge with irradiated leukaemic cells was unnecessary. Because of the high cure rate obtained, the very regular response pattern and the non-pathogenicity, the bacterium Micrococcus lysodeikticus would seem a promising new candidate for chemo-immunotherapeutic antitumour strategies.


Br. J. Cancer (1981) 43, 201

### SUCCESSFUL IMMUNOTHERAPY WITH MICROCOCCUS, BCG OR RELATED POLYSACCHARIDES ON L1210 LEUKAEMIA AFTER BCNU CHEMOTHERAPY

R. VERLOES*, G. ATASSI†, P. DUMONT† AND L. KANAREK*‡

*From the *Laboratorium voor Chemie der Proteïnen, Vrije Universiteit Brussel, Instituut voor Moleculaire Biologie, Paardenstraat 65, B-1640 Sint-Genesius-Rode, and †Service de Médecine Interne et Laboratoires d'Investigation Clinique Henri Tagnon (Section de Chimiothérapie Expérimentale), Centre des Tumeurs de l'Université Libre de Bruxelles, Belgium*

Received 18 February 1980 Accepted 10 October 1980

**Summary.—The experiments aimed at evaluating the optimal parameters in the chemo-immunotherapeutic treatment of the L1210 lymphoid leukaemia grafted to [female BALB/c ($H_2^d$) × male DBA/2 ($H_2^d$)]$F_1$ hybrid mice, hereafter referred to as CDF1 mice.**

***In vitro* irradiation of leukaemic ascites cells by X- or γ-rays and subsequent inoculation in mice showed that optimum immunogenicity is radiation dose-dependent. Grafting mice with $10^7$ leukaemic ascites cells irradiated at optimum dose (80 GyX- or γ-rays) delays mortality of the animals when challenged later with untreated L1210 cells, but is unable to cure mice. By contrast, specific immunoprophylaxis induced by Micrococcus, complement-triggering polysaccharides or BCG and irradiated leukaemic cells was able to protect mice against grafts of $10^4$ L1210 cells. The i.p. route was notably superior to the i.v. route.**

**When mice bearing advanced L1210 tumour were treated by chemotherapy (12 mg/kg of BCNU) on Day 6·5 after grafting $10^4$ L1210 cells and subsequently treated by immunotherapy, a very high percentage (up to 90%) of mice with $10^8$ leukaemic cells could be cured by repeated 1mg injections of bacterium or polysaccharide, and challenge with irradiated leukaemic cells was unnecessary.**

**Because of the high cure rate obtained, the very regular response pattern and the non-pathogenicity, the bacterium *Micrococcus lysodeikticus* would seem a promising new candidate for chemo-immunotherapeutic antitumour strategies.**

THE BCG STRAIN of *Mycobacterium bovis* has become a popular agent for immunotherapy of cancer. However, the general use of BCG is made difficult because of many disadvantages: BCG can cause ulceration, pyrexia, abnormal liver function, tuberculosis and, in a few reported cases, death or even enhancement of tumour growth (Hunt *et al.*, 1973; Spark *et al.*, 1973; Mansell & Krementz, 1973).

*Micrococcus lysodeikticus* is a non-pathogenic and easy-to-eliminate (substrate of lysozyme) Gram-positive bacterium, that elicits the production of large amounts of antibodies of restricted heterogeneity and clonal dominance in rabbits (Van Hoegaerden *et al.*, 1975) and mice (Verloes *et al.*, 1977*a*; *b*). Since antimicrococcus antibodies are directed against carbohydrates (Wikler, 1975) and bind to certain lymphocytes (Verloes & Kanarek, 1976; De Baetselier *et al.*, 1977) and to several tumour-cell types (L1210 lymphoid cells, Ehrlich carcinoma cells) (Verloes *et al.*, 1976) but not to erythrocytes (Verloes *et al.*, 1979*a*), they are believed to act on cells in a similar way to the lectins but with greater discrimination and less

‡ To whom requests for reprints and correspondence should be addressed.

toxicity for normal cells (Verloes *et al.*, 1976). The interaction of antimicrococcus antibodies with receptors on neoplastic cells was confirmed by others, and a cell-cycle-stage dependency was established (Grooten & Hamers, 1979).

Although a single injection of Micrococcus suspended in incomplete Freund adjuvant does not display an adjuvant effect for protein antigens (Kotani *et al.*, 1975), we have demonstrated that multiple Micrococcus injections, like live BCG injections, elicit a comparable number of plaque-forming cells to sheep erythrocytes, whereas Micrococcus-treated mice showed markedly less toxicity than BCG-treated animals (Verloes *et al.*, 1979*b*). We have also previously demonstrated that *Micrococcus lysodeikticus* is able to activate complement by the alternative pathway (Verloes *et al.*, 1977*b*) and the importance of this phenomenon in inflammation is well documented (Bianco *et al.*, 1976; Ward, 1967, Schorlemmer *et al.*, 1976). In an attempt to maximize the chemotherapeutic response to antitumour agents by immunotherapy, this study was undertaken to compare therapeutic values of Micrococcus with the activity generated by structurally related complement-triggering polysaccharides and BCG. Immunological monitoring after cytotoxic chemotherapy is likely to be highly dependent on the immune status of the host, and subsequently correlated with the drug used and the chemotherapeutic regimen. Furthermore, since it was shown that chemoimmunotherapy of L1210 leukaemia by a drug (cyclophosphamide) and an antigen (L1210 cells) depending on the protocol, may be either non-significant (Mathé *et al.*, 1977) or effective (Kataoka *et al.*, 1978), special attention was paid to determine the experimental parameters that yield an optimal antitumour response.

### MATERIAL AND METHODS

*Micrococcus lysodeikticus* (*ML*), *cell wall of ML*, *cell-wall-conjugated chitin and chitin* were prepared as earlier described (Verloes *et al.*, 1976).

*Bacillus Calmette–Guérin.*—Vials containing 100 mg of lyophilized *Bacillus Calmette–Guérin* (BCG-SP) for scarification were purchased from the Pasteur Institute, Brussels. After reconstitution with the appropriate diluent and further dilution in Hanks' balanced salt solution (HBSS) the suspension was kept at 4°C and used within 2 weeks.

*Zymosan A.*—The cell wall of *Saccharomyces cerevisiae* (yeast) was commercially available (Lot No. 34C–2650—Sigma Company). Insoluble cell walls were boiled for 1 h in 0·9% NaCl solution, washed ×3 with PBS and centrifuged at 3000 *g* for 20 min.

*Inulin.*—The $\beta$(2–1) polyfructoside inulin (Lot No. 519 903) was obtained from J.T. Baker Company.

*Dextran sulphate* (sodium salt).—The material used was $\alpha$(1–4) polysaccharide with approximate mol. wt of 500,000, and was purchased from Pharmacia Fine Chemicals (Lot No. 7126).

*BCNU.*—Vials containing 100 mg of 1,3 bis-(2-chloroethyl)-1-nitrosourea, named BCNU (NSC-409962), were obtained from the National Cancer Institute, Bethesda, Maryland, U.S.A. After reconstitution with the appropriate diluent and further dilution in ice-chilled HBSS, the solution was injected within 2 h.

*Animals.*—Female [female BALB/c ($H_2^d$) × male DBA/2 ($H_2^d$)] $F_1$ hybrid mice (CDF1) were purchased from Charles River Breeding Laboratories, Calco, Italy. Those mice were stored 3 weeks in an isolation room and used before they were 10 weeks old. Animals weighing 19–23 g were used.

*Tumour.*—L1210 leukaemia was originally induced in female DBA/2 mice with methylcholanthrene in ether (Law *et al.*, 1949). The L1210 leukaemia, obtained from Dr A. Bogden (Mason Research Institute, Worcester, Mass., U.S.A.), was maintained in ascitic form by weekly transfer in DBA/2 mice. Animals were injected i.p. to obtain the ascitic form of leukaemia, or i.v. to obtain the blood form of leukaemia. Animals were randomized into test and control groups.

Mean survival time (MST) of treated and control mice as well as the doses and timing of each experiment are specified in the tables and figures. Mice still tumour-free on the 90th day after tumour grafting were considered as long-term survivors and eliminated from evaluation of the mean survival time. No relapses after Day 90 were seen.

### RESULTS

### *Effect of irradiation source and dose on lethality and immunogenicity of L1210 cells*

L1210 cells grown as ascites in DBA/2 mice were aspirated from the mouse peritoneal cavity 7 days after grafting $10^4$ cells i.p. These cells were diluted in HBSS and irradiated at different doses (0, 40, 80, 120 and 170 Gy) by a $\gamma$-ray-emitting $^{60}$Co Siemens apparatus at a dose rate of 3·55 Gy/min at 200 kV. $10^7$ irradiated cells were grafted i.p. to intact CDF1 mice and the mortality was recorded. Whereas mice challenged with unirradiated cells died 6·50 days ± 0·55 later, mice injected with 40Gy-irradiated L1210 cells died 12·83 ± 0·75 days after injection. No mortality was seen with the other grafts. When surviving mice were rechallenged 30 days later with $10^4$ viable leukaemic L1210 cells, we found a 92, 18 and 7% increase in MST over control mice respectively for the 80, 120 and 170Gy schedules, as seen in Table I. In order to compare the effect of the irradiation source on lethality and immunogenicity of L1210 cells, the same experiment was performed using an Euratom X-ray apparatus (250 kV, 12 mA, 0·95 Gy/min). Whereas $10^7$ unirradiated cells killed mice 5·50 days ± 0·71 after grafting, 40Gy-irradiated leukaemic cells killed mice 14·90 days ± 1·91 after challenge. No mortality was seen with the other grafts. When survivors were rechallenged with $10^4$ cells i.p., we found a 53, 48 and 27% increase in MST over controls respectively, as seen in Table I. Long-term survivors (> 90 days) were never recorded. This leads us to conclude that the optimal irradiation dose (80 Gy) may exist irrespective of irradiation source.

### *Specific anti-L1210 immunoprophylaxis by a combination of irradiated leukaemic L1210 cells and bacterium or polysaccharide*

To evoke a specific immune response, randomized intact CDF1 mice were immunized by grafting $10^7$ irradiated (80 Gy $\gamma$-rays) L1210 ascites cells simultaneously with 1 mg of bacterium or polysaccharide either i.p. or i.v. Two months later, immunized mice received a transplant of $10^4$ viable L1210 cells according to the immunization route. As shown in Table

TABLE I.—*Effect of irradiation dose on lethality and immunogenicity of L1210 leukaemic cells*

| Irradiation of L1210 cells | | MST (days) ± s.d.† after grafting $10^7$ irr. cells | MST (days) ± s.d.† rechallenge with $10^4$ cells | ILS§ (%) | % of long-term survivors (Day 90) |
|---|---|---|---|---|---|
| Source | Dose (Gy) | | | | |
| X-rays | 0 | 5·50 ± 0·71 | 10·54 ± 0·66 (controls) | 0 | 0 |
| | 40 | 14·90 ± 1·91 | — | — | |
| | 80 | — | 16·14 ± 1·34* | 53 | 0 |
| | 120 | — | 15·60 ± 3·89* | 48 | 0 |
| | 170 | — | 13·43 ± 7·91 | 27 | 0 |
| $\gamma$-rays | 0 | 6·50 ± 0·55 | 9·50 ± 0·71 (controls) | 0 | 0 |
| | 40 | 12·83 ± 0·75 | — | — | |
| | 80 | — | 18·25 ± 15·5 | 92 | 0 |
| | 120 | — | 11·25 ± 1·26 | 18 | 0 |
| | 170 | — | 10·16 ± 0·41 | 7 | 0 |

Leukaemic L1210 cells grown as ascites in DBA/2 mice were irradiated at different doses and $10^7$ cells were grafted i.p. in CDF1 mice.

Surviving mice were rechallenged one month later with $10^4$ L1210 cells and MST of rechallenged mice was compared to that of tumour-challenged control mice.

* Significant at $P < 0.001$ (Student–Fisher $t$ test).

† For mice dying before 90 days after grafting cells.

‡ 10–20 mice in each experiment.

§ ILS = increase in lifespan of treated mice over control mice.

TABLE II.—*Specific anti-L1210 immunoprophylaxis by prevaccination with irradiated cells and micrococcus, BCG or related polysaccharides*

| Treatment: Irradiated cells | Treatment: Agent | Route (treatment + transplant) | % of long-term survivors (Day 90) | MST (days) ± s.d. | ILS* (%) | t test |
|---|---|---|---|---|---|---|
| + | BCG | i.v. | 0 | 12·00 ± 2·31 | 17 | NS |
| + | Micrococcus | i.v. | 12·5 | 11·14 ± 0·69 | 9 | NS |
| + | Zymosan | i.v. | 0 | 10·43 ± 0·53 | 2 | NS |
| + | Chitin | i.v. | 0 | 12·50 ± 4·23 | 22 | NS |
| + | Inulin | i.v. | 14·0 | 8·67 ± 3·72 | 0 | NS |
| + | Dextran sulphate | i.v. | 14·0 | 10·83 ± 0·98 | 6 | NS |
| + | (controls) | i.v. | 0 | 14·14 ± 5·81 | 38 | NS |
| + | BCG | i.p. | 86·0 | 10·00 ± 0·0 | 0 | NS |
| + | Micrococcus | i.p. | 71·0 | 11·00 ± 0·0 | 8 | NS |
| + | Zymosan | i.p. | 86·0 | 11·00 ± 0·0 | 8 | NS |
| + | Chitin | i.p. | 0 | 10·33 ± 0·52 | 1 | NS |
| + | Inulin | i.p. | 57·0 | 12·33 ± 2·52 | 21 | NS |
| + | Dextran sulphate | i.p. | 14·0 | 12·33 ± 3·01 | 21 | NS |
| + | (controls) | i.p. | 17·0 | 11·20 ± 0·45 | 10 | NS |
| – | (controls) | i.p. | 0 | 10·22 ± 0·44 | 0 | — |

Intact CDF1 mice were immunized by grafting $10^7$ irradiated (80 Gy, $\gamma$-rays) *in vivo*-grown L1210 cells and 1 mg of different agents, 2 months before transplantation of $10^4$ leukaemic L1210 cells; 10–20 mice per expt.

* ILS = increase in lifespan of treated (but tumour-bearing and dying before 90 days) mice over control mice.

II, the i.p. route generated a more effective immunoprotection. Our data also indicate that immunoresponse leads to an all-or-none reaction; it is able to induce long-term survivors but unable to prolong significantly mean survival of the other immunized and tumour-bearing mice. This fact does not weaken the results, especially since the number of animals surviving to Day 90 is very high. There were slight differences in immunoprotection rates for zymosan (86%), Micrococcus (71%) and BCG (86%). From these experiments, we also learned that the i.p. route definitely yielded better results, and consequently was used in the following chemo-immunotherapeutic trial.

*Chemo-immunotherapy: drug design and experimental control*

These experiments were conducted to maximize the chemotherapeutic response of an antitumour drug therapy by immunotherapy. We concentrated our efforts on advanced (terminal) disease for which treatment is initiated several days before the expected death day of controls. For that purpose, CDF1 mice were given i.p. $10^4$ L1210 cells on Day 0 and the tumour was allowed to grow and spread. On Day 6·5, when the tumour burden is considered to reach $10^8$ cells (Cantrell *et al.*, 1976) and treatment by bacterial immunoadjuvant only (BCG or ML) proved to be unsuccessful, mice received one single i.p. injection of BCNU (12 mg/kg) and were subsequently treated by immunotherapy on Day 8, either by $10^7$ irradiated (80 Gy $\gamma$-rays) *in vivo*-grown L1210 cells combined with 1 mg of bacterium or polysaccharide, or by 1 mg of bacterium or polysaccharide alone. This experimental protocol for chemotherapy was adopted from Cantrell *et al.* (1976). All untreated control mice died after 10·22 days ± 0·44, whereas mice treated by chemotherapy died after 19·80 days ± 4·34 and mice treated by chemotherapy and $10^7$ irradiated cells died after 20·80 days ± 5·07. No long-term survivors were recorded.

As seen in Table III, a single administration of bacteria or some complement-triggering polysaccharides induces an appreciable % of long-term survivors (> 90 days) which are freed from $10^8$ leukaemic L1210 cells.

Looking at these data, we are tempted to believe that the administration of

TABLE III.—*Effect of a single injection of Micrococcus, BCG and related polysaccharides in specific or non-specific immunotherapy of L1210 leukaemia after BCNU chemotherapy*

| Immunotherapy: Irradiated cells | Immunotherapy: Agents | MST (days) ± s.d. | % of long-term survivors (Day 90) | ILS (%) | *t* test |
|---|---|---|---|---|---|
| + | BCG | 26·71 ± 10·36 | 25 | 35 | NS |
| + | Micrococcus | 27·89 ± 5·11 | 10 | 41 | $P < 0·001$ |
| + | Zymosan | 30·20 ± 16·39 | 50 | 52 | NS |
| + | Chitin | 28·00 ± 14·68 | 20 | 41 | NS |
| + | Inulin | 24·29 ± 9·81 | 30 | 23 | NS |
| + | Dextran sulphate | 16·33 ± 2·55 | 10 | 0 | NS |
| + | Cell-wall chitin | 22·60 ± 4·70 | 0 | 14 | NS |
| + | Control (chemotherapy only) | 20·80 ± 5·07 | 0 | 5 | NS |
| − | BCG | 26·43 ± 8·94 | 12·5 | 33 | NS |
| − | Micrococcus | 22·90 ± 5·34 | 0 | 16 | NS |
| − | Zymosan | 28·00 ± 9·54 | 50 | 41 | NS |
| − | Chitin | 29·13 ± 7·26 | 11 | 47 | NS |
| − | Inulin | 24·29 ± 5·12 | 30 | 23 | NS |
| − | Dextran sulphate | 19·83 ± 7·17 | 14 | 0 | NS |
| − | Cell-wall chitin | 22·80 ± 9·68 | 0 | 15 | NS |
| − | Control (chemotherapy only) | 19·80 ± 4·34 | 0 | 0 | NS |
| | No immunotherapy and no chemotherapy | 10·22 ± 0·44 | 0 | — | — |
| + | No chemotherapy | 10·20 ± 0·42 | 0 | — | — |

Intact CDF1 mice received an i.p. transplant of $10^4$ leukaemic L1210 cells on Day 0. They then received a single i.p. injection of BCNU (12 mg/kg) on Day 6·5, followed by immunotherapy on Day 8 and an injection of $10^7$ irradiated (80 Gy $\gamma$-rays) *in vivo*-grown L1210 cells combined with 1 mg bacterium or polysaccharide alone; 10–20 mice per expt.

The increase in mean lifespan of mice treated by chemo-immunotherapy over mice treated by chemotherapy is expressed as a percentage.

irradiated ascites cells yields better results. Using the same chemo-immunotherapeutic protocol, we have investigated next whether repeated administration of complement triggers or bacteria on Days 9, 10, 11, and 12 or on Days 11, 14, 17 and 20 might increase the number of long-term survivors (mice cured on Day 90). By combining chemotherapy with nonspecific (no irradiated cells) immunotherapy, we are able, except for chitin, to cure 30–90% of mice with $10^8$ leukaemic cells by 1mg injections on Days 8, 9, 10, 11 and 12, and 40–90% of mice by injections on Days 8, 11, 14, 17 and 20. In both treatment schedules, the administration of Micrococcus yielded the highest scores of long-term survivors (90%), as illustrated in Figs 1 and 2.

In a final set of experiments, we have investigated whether inoculation of $10^7$ leukaemic cells irradiated at optimal dose (80 Gy $\gamma$-rays) could improve the chemo-immunotherapeutic schedule described above. As illustrated in Figs 3 and 4, we were able to induce long-term survivors by this combination of chemotherapy with immunotherapy in 30–90% of the animals injected on Days 8, 9, 10, 11 and 12 and in 30–90% of those injected on Days 8, 11, 14, 17 and 20. By contrast, mice treated by chemotherapy only died after 26·12 days ± 9·72, yielding 13% of long-term survivors (2/15), whereas mice treated by chemotherapy and $10^7$ irradiated cells survived 25·67 days ± 5·26 with also 13% of long-term survivors (2/15) (the latter data do not appear in Figs 1–4).

We conclusively demonstrated that Micrococcus, zymosan A and inulin applied after BCNU therapy yielded an appreciably greater percentage ($P < 0·005$) of cured mice than leukaemic mice given BCNU chemotherapy only. The efficiency of BCG chemo-immunotherapy critically depended on the immunotherapeutic protocol. By contrast, chemo-immunotherapy performed with cell-wall chitin, dextran sulphate or chitin was less effective or ineffective.

FIG. 1.

FIGS 1–4.—Effect of repeated administration of Micrococcus BCG and related polysaccharides in non-specific chemo-immunotherapy of L1210 leukaemia. Intact CDF1 mice received an i.p. transplant of $10^4$ leukaemic L1210 cells on Day 0. They were then treated by a single i.p. injection of BCNU (12 mg/kg) on Day 6·5, and treated by immunotherapy as an i.p. injection of 1 mg on Days 8, 9, 10, 11 and 12 (Fig. 1) or on Days 8, 11, 14, 17 and 20 (Fig. 2). Other mice were treated by immunotherapy on Day 8 and received an i.p. injection of $10^7$ irradiated (80 Gy $\gamma$-rays) *in vivo*-grown L1210 cells combined with 1 mg bacterium or polysaccharide. Treatment was continued by i.p. 1mg injections on Days 9, 10, 11 and 12 (Fig. 3) or on Days 11, 14, 17 and 20 (Fig. 4). Statistical evaluations were determined by $\chi^2$ distribution for difference from mice given BCNU only. NS = not significant.

FIG. 2.

FIG. 3.

FIG. 4.

### DISCUSSION

Many antigenic tumours such as L1210 leukaemia are poorly immunogenic (Cantrell *et al.*, 1976) and require stimuli to elicit immune responses (Glynn *et al.*, 1963). As more effective stimuli, we have used BCG, Micrococcus and structurally related complement-triggering polysaccharides. Previous studies indicated that pretreatment of mice with those agents alone was insufficient to provide immunoprotection against tumour grafting (Verloes *et al.*, 1978). We have therefore compared the effect of different doses of X- or $\gamma$-rays on lethality of *in vivo*-grown (ascites) L1210 cells and their capability of inducing immunoprotection. Our results clearly demonstrate an optimal irradiation dose (80 Gy); the mortality of mice challenged with radiated cells was delayed but no cures were recorded. However, specific antitumour immunoprophylaxis induced by preimmunization with irradiated leukaemic cells and bacteria or polysaccharide was more effective when we used the i.p. than the i.v. route, and yielded a high percentage of mice cured on the 90th day after tumour grafting. Since it was shown that L1210 leukaemia growth is accompanied by tumour-mediated immunosuppression (Huget *et al.*, 1977), it would be interesting to investigate whether the kinetics of immunosuppression affects similarly i.v. or i.p. challenged mice. Although chloro-ethyl-nitrosoureas constitute an established class of important antitumour agents, repeated administration of BCNU to human patients may produce a dramatic marrow toxicity (Schein *et al.*, 1978). Therefore, applying successful immunotherapy after effective chemotherapy instead of maintenance chemotherapy, is one possibility for counteracting long-lasting drug toxicity in humans and mice, and reducing the probability of *in vivo* selection of drug-resistant neoplastic cell clones, thus offering a chance of cure. When tumour-bearing mice were treated (once) by BCNU chemotherapy 6·5 days after inoculation of $10^4$ leukaemic cells and treated (once) on Day 8 by immunotherapy, a considerable percentage of long-term survivors was recorded. Our best results were obtained with zymosan (50% survivors). However, administration of Micrococcus yielded a very low percentage of surviving mice (10%). This might be due to the easy destruction and elimination of the complement-triggering bacterium Micrococcus (Verloes *et al.*, 1977*b*) by the polymorphonuclear leucocytes and the subsequent exocytosis of lysosomal enzymes (*i.e.* lysozyme) by these cells (Schorlemmer *et al.*, 1976). Probably this also accounts for the observation that repeated 1mg Micrococcus injections enhance 5-fold the number of direct plaque-forming cells to sheep erythrocytes, whereas one injection displays no effect (Verloes *et al.*, 1979*b*). By using low doses of BCNU with nonspecific (without irradiated cells) immunotherapy, we were able to cure up to 90% of mice bearing $10^8$ leukaemic cells by repeated 1mg injections of bacteria or polysaccharides. Highest survival scores were obtained with *Micrococcus lysodeikticus*. Furthermore, although the data presented do not demonstrate clearly the superiority of Micrococcus over zymosan or inulin chemo-immunotherapy, we must mention that antimicrococcus antibodies selectively recognize epitopes on tumour cells such as L1210 cells (Verloes *et al.*, 1976) and that repeated 1mg injections of BCG to intact mice produced a considerable loss of weight which did not occur after *Micrococcus lysodeikticus* (Verloes *et al.*, 1979*b*). The fact that the therapeutic values of Micrococcus and zymosan A were rather schedule-independent, whereas values obtained with BCG were critically influenced by timing and antigen presentation, strengthens the interest in Micrococcus. It is also obvious that repeated immunological treatments are more effective than a single administration, and that rechallenge with irradiated leukaemic cells is not strictly required for this working schedule. Very probably, effective cytotoxic chemotherapy provided immunocompetent cells with immunogenic material, and the immune system was subsequently triggered by complement-activating polysaccharides or bacteria. In this perspective, it is worth mentioning that a similar prevailing condition can be created by intratumour cytotoxic chemotherapy (followed by intratumour immunotherapy) of inoperable cancers where it is impossible to obtain antigens.

The high cure rates obtained, the regular therapeutic response (at least as good or better than with BCG and zymosan) the absence of toxicity and the non-pathogenicity of *Micrococcus lysodeikticus* promote this easy-to-eliminate bacterium (substrate of lysozyme) as an attractive candidate for new chemo-immunotherapeutic antitumour strategies.

The authors express their gratitude to Professor P. Stryckmans for discussions and specific constructive criticisms.

They thank Dr R. Regnier and Professor J. Urbain for the use of $^{60}$Co and X-ray apparatus.

They are also grateful to Dr O. Yoder who has supplied the BCNU samples.

This work was supported by Contract No. N01-CM-53840 entered into with the National Cancer Institute, Bethesda, Maryland, U.S.A., and by a special grant from the Belgian Government (Fonds voor Onderling Overlegde Akties) and the A.S.L.K.-C.G.E.R. Cancer Fund.

### REFERENCES

BIANCO, C., EDEN, A. & COHN, Z. A. (1976) The induction of macrophage spreading: Role of coagulation factors and the complement system. *J. Exp. Med.*, **144**, 1531.

CANTRELL, J. L., KILLION, J. J. & KOLLMORGEN, G. M. (1976) Correlations between humoral immunity and successful chemo-immunotherapy. *Cancer Res.*, **36**, 3051.

DE BAETSELIER, P., GROOTEN, J., VAN DE WINKEL, M. & HAMERS, R. (1977) Probing lymphoid systems with antimicrococcus antibodies. I. Effect on lymphocyte responses to mitogens *in vitro*. *Internat. Physiol. Biochem.*, **85**, 161.

GLYNN, J. P., HUMPHREYS, S. R., TRIVERS, G., BIANCO, A. R. & GOLDIN, A. (1963) Studies on immunity to leukaemia L1210 in mice. *Cancer Res.*, **23**, 1008.

GROOTEN, J. & HAMERS, R. (1979) Recognition of a cell cycle dependent membrane marker in mouse lymphoid lines by anti-Micrococcus antibodies. *VIIth Meeting of Int. Soc. Oncodevel. Biol. Med.*, Surrey, U.K.

HUGET, R. P., FLAD, H.-D. & OPITZ, H. G. (1977) Secretion of *in vitro* primary immune response by L1210 cells and their culture supernatant. *Cell. Immunol.*, **29**, 210.

HUNT, J. S., SILVERSTEIN, M. J. & SPARKS, F. C. (1973) Granulomatous hepatitis: A complication of BCG therapy. *Lancet*, ii, 820.

KATAOKA, T., KOBAYASHI, H. & SAKURAI, Y. (1978) Potentiation of Concanavalin A-bound L1210 vaccine *in vivo* by chemo-therapeutic agents. *Cancer Res.*, **38**, 1202.

KOTANI, S., NARITA, T., STEWART-TULL, D. E. S. & 4 others (1975) Immuno-adjuvant activities of cell walls and their water-soluble fractions prepared from various Gram-positive bacteria. *Biken J.*, **18**, 77.

LAW, L. W., DUNN, T. B., BOYLE, P. J. & MILLER, J. H. (1949) Observations on the effect of a folic-acid antagonist on transplantable lymphoid leukaemia in mice. *J. Natl Cancer Inst.*, **10**, 179.

MANSELL, P. W. & KREMENTZ, E. T. (1973) Reactions to BCG. *J. Am. Med. Ass.*, **226**, 1570.

MATHE, G., HALLE-PANENKO, O. & BOURUT, C. (1977) Interspersion of cyclophosphamide and BCG in the treatment of L1210 leukaemia and Lewis tumour. *Eur. J. Cancer*, **13**, 1095.

SCHEIN, P. S., BULL, J. M., DOUKAS, D. & HOTH, D. (1978) Sensitivity of human and murine hematopoietic precursor cells to 2-[3-(2-chloroethyl)-3-nitroso-ureido]-D-glucopyranose and 1,3-bis(2-chloroethyl)-1-nitroso-urea. *Cancer Res.*, **38**, 257.

SCHORLEMMER, H. V., DAVIES, P. & ALLISON, A. C. (1976) Ability of complement components to induce lysosomal enzyme release from macrophages. *Nature*, **261**, 48.

SPARK, S. F. C., SILVERSTEIN, M. J. & HUNT, J. S. (1973) Complications of BCG immunotherapy in patients with cancer. *N. Engl. J. Med.*, **289**, 827.

VAN HOEGAERDEN, M., WIKLER, M., JANSSENS, R. & KANAREK, L. (1975) Antibodies to *Micrococcus lysodeikticus*: Restricted structural heterogeneity in hyperimmunized rabbits. *Eur. J. Biochem.*, **53**, 19.

VERLOES, R. & KANAREK, L. (1976) Interactions of the lectins PHA, Con A and antimicrococcus with blood cells of different species and Ehrlich carcinoma cells. *Arch. Intern. Physiol. Biochem.*, **85**, 418.

VERLOES, R., ATASSI, G. & KANAREK, L. (1976) Antitumour immunoprotection by an immunobacterial lectin-approach. *Eur. J. Cancer*, **12**, 877.

VERLOES, R., MACHTELINCKX, V., THEUNISSEN, J. & KANAREK, L. (1977*a*) The immune response of mice to *Micrococcus lysodeikticus*: Evidence for serum-mediated immunoregulation. *Biochem. Soc. Trans.*, **5**, 1156.

VERLOES, R., DE RIDDER, M. & KANAREK, L. (1977*b*) Biochemical properties that accompany the production of homogeneous antibody response: a general mechanism hypothesis. *Biochem. Soc. Trans.*, **5**, 1158.

VERLOES, R., ATASSI, G., DUMONT, P. & KANAREK, L. (1978) Influence of Micrococcus, BCG and related polysaccharides on the proliferation of the L1210 leukaemia. *Br. J. Cancer*, **38**, 599.

VERLOES, R., ATASSI, G. & KANAREK, L. (1979*a*) Comparison between the *in vitro* interaction of lectins (PHA and Con A) and antimicrococcus antibodies on normal and malignant cells. *Eur. J. Cancer*, **15**, 1439.

VERLOES, R., HUYGEN, K., BECKERS, E., ATASSI, G. & KANAREK, L. (1979*b*) Effect of Micrococcus, BCG and structurally related polysaccharides on the adjuvanticity to a T-cell dependent antigen. *Arch. Internat. Physiol. Biochem.*, **87**, 861.

WARD, P. A. (1967) A plasmin split fragment of $C_3$ as a new chemotactic factor. *J. Exp. Med.*, **126**, 189.

WIKLER, M. (1975) Isolation and characterization of homogeneous rabbit antibodies to *Micrococcus lysodeikticus* with specificity to the peptidoglycan and the glucose-N-acetyl-mannosaminuronic acid polymer. *Z. Immun.-Forsch.*, **149**, 193.